# A Simple Metallothionein-Based Biosensor for Enhanced Detection of Arsenic and Mercury

**DOI:** 10.3390/bios7010014

**Published:** 2017-03-13

**Authors:** Gordon W. Irvine, Swee Ngin Tan, Martin J. Stillman

**Affiliations:** 1 Department of Chemistry, The University of Western Ontario, 1151 Richmond St, London, ON N6A 5b7 Canada; girvine@uwo.ca; 2 Natural Sciences and Science Education Academic Group, Nanyang Technological University, 1 Nanyang Walk, 637616 Singapore, Singapore; sweengin.tan@nie.edu.sg

**Keywords:** paper-based biosensor, metallothionein, arsenic, mercury, green chemistry, screen-printed electrode

## Abstract

Metallothioneins (MTs) are a family of cysteine-rich proteins whose biological roles include the regulation of essential metal ions and protection against the harmful effects of toxic metals. Due to its high affinity for many toxic, soft metals, recombinant human MT isoform 1a was incorporated into an electrochemical-based biosensor for the detection of As^3+^ and Hg^2+^. A simple design was chosen to maximize its potential in environmental monitoring and MT was physically adsorbed onto paper discs placed on screen-printed carbon electrodes (SPCEs). This system was tested with concentrations of arsenic and mercury typical of contaminated water sources ranging from 5 to 1000 ppb. The analytical performance of the MT-adsorbed paper discs on SPCEs demonstrated a greater than three-fold signal enhancement and a lower detection limit compared to blank SPCEs, 13 ppb for As^3+^ and 45 ppb for Hg^2+^. While not being as low as some of the recommended drinking water limits, the sensitivity of the simple MT-biosensor would be potentially useful in monitoring of areas of concern with a known contamination problem. This paper describes the ability of the metal binding protein metallothionein to enhance the effectiveness of a simple, low-cost electrochemical sensor.

## 1. Introduction

Toxic metals are found naturally in the earth’s crust at a very wide range of concentrations and are present in many environments. The inherent toxicity of these metals and their bioavailability influence the extent to which they pose problems for human health as well as plant and animal life. Two species with significant effects on human health are As^3+^ and Hg^2+^ [1,2]. Mercury contamination, which has been well documented in Japan, as well as in other countries including Canada, has caused thousands of cases of Minamata disease [3]. Clearly, contamination from industry, agriculture and natural deposition must be monitored in a cost-effective and rapid method to prevent future cases [4]. Hg^2+^ is the most common speciation of mercury in aquatic systems owing to its biotransformation into an organic species [5]. Metallothionein (MT) has been shown to bind mercury with a high affinity and form stable complexes with a variety of species [6,7,8,9]. However, MT does not bind specifically to any one metal due to its fluxional nature and lack of defined binding sites that would discriminate against certain metal co-factors [10,11].

Arsenic, in particular As^3+^, is present at chronic levels in drinking water in many parts of the world but is not evenly distributed among sources [12]. Therefore, detection and quantification of arsenic in potable water and even water used for irrigation that can bioaccumulate in crops, is vital in assessing the security of various water sources [13,14].

Populations that are affected by significant arsenic contamination of their water supply tend to be poor and have limited access to expensive and centrally located testing facilities [15,16]. A cheap, portable, reliable and easy-to-use method to detect arsenic would help in monitoring contamination levels in poor, rural areas of South and Southeast Asia. The method must have a low detection limit due to the high toxicity of arsenic and mercury. Even low concentrations (<50 ppb) of arsenic can have serious chronic effects [17,18]. Biosensors are a broad category of sensors based on biological materials that may meet these criteria of being cheap, portable and reliable. 

Biosensors containing DNA, enzymes and metal-binding proteins are promising tools with which to obtain real-time, in-situ data for heavy metal contamination [19,20]. MTs are a family of cysteine-rich, metal-binding proteins that bind soft metals like mercury with a particularly high affinity [21,22,23]. Hg^2+^ has been shown to have higher affinity for MTs than other Hg-species [24,25].

At low pH, MTs are able to bind strongly to As^3+^ although the reaction is slower than with native metals (zinc or copper) at neutral pH [7,26,27]. Cd-MT and Zn-MT demetalate below pH 3.5 [28] leaving the metal-free apo-MT available for the coordination of arsenic species [29]. The unique properties of MT make it an excellent candidate for increasing the sensitivity of electrochemical sensors, and efforts have been made to create MT-modified electrodes for metal sensing [30,31,32,33]. The success of many of these devices is limited due to the time consuming preparation and expense required for other essential components, such as MT-specific antibodies [34] or reducing agents [35]. In this study, our goal was to develop a low-cost, environmentally friendly biosensor using MT adsorbed onto paper discs and placed on screen printed carbon electrodes (SPCEs) for the detection of arsenic and mercury. 

## 2. Materials and Methods

### 2.1. Reagents and Instrumentation

All reagents used were of analytical grade. Electrochemical characterizations and measurements were performed using a four-channel system (eDAQ QuadStat, e-Corder 8 and Echem software, eDAQ Europe, Warsaw, Poland). SPCEs (DRP-110) and the boxed connector for SPEs (DRP-DSC) were purchased from DropSens (Asturias, Spain). The working electrode was carbon while the reference and counter electrodes were Ag/AgCl and a carbon ring, respectively. Circular paper discs were cut from Grade 1 filter paper (Whatman Asia Pacific Pte Ltd., Singapore). Standard solutions of 1000 ppm As^3+^ and Hg^2+^ were diluted with 18 MΩ ultrapure water obtained from a Millipore Alpha-Q water system (Bedford, MA, USA) to final concentrations ranging from 1 ppm to 5 ppb. Data were plotted on Microsoft Excel and refined using ORIGIN (Northhampton, MA, USA).

### 2.2. Recombinant Protein Preparation

Recombinant human metallothionein 1a (MGKAAAACSC ATGGSCTCTG SCKCKECKCN SCKKCC SCCPMSCAKC AQGCVCKGAS EKCSCCK KAA AA) was expressed with an S-tag in BL21 *E. coli* cells, as described in detail elsewhere [36]. In brief, cells containing the plasmid for the full protein (βα-MT1a) were plated on to growth media containing kanamycin from a stock culture stored at −80 °C and grown for 16 h at 37 °C. The grown cells were then inoculated into 4 × 1 L broth cultures enriched with 50 μL of 1 M cadmium sulfate and incubated in a shaker for 4 h until the OD_600_ absorbance was 0.8. Isopropyl β-d-1-thiogalactopyranoside (IPTG) was then added to induce expression of MT and 30 min later 150 μL of 1 M cadmium sulfate solution was added to the broth. The cells were collected 3.5 h after induction, centrifuged and stored at −80 °C.

The recombinant cells were lysed using a cell disruptor (Constant Systems, Daventry, UK) shot at 20 k psi. From there, the cell lysate was centrifuged for 1 h to pellet out cellular debris. The supernatant was filtered and loaded on to a GE healthcare SP ion exchange column with a total volume of 10 mL. The columns were washed with pH 7.4 10 mM Tris(tris-hydroxymethyl-aminomethane) buffer for approximately 2 h to remove loosely bound proteins and other organic compounds. MT was eluted using an increasing gradient of 1 M NaCl + 10 mM Tris buffer at pH 7.4. The eluted MT was concentrated down to <20 mL and lyophilized for storage and transport. 

The lyophilized MT was reconstituted in 10 mM ammonium formate buffer and buffer exchanged to remove excess salt. The MT solution was checked for oxidation using UV-Vis spectroscopy and ESI mass spectrometry to ensure the lyophilisation process did not cause oxidation of the MT thiols or loss of bound metals.

### 2.3. Disc Preparation and Electrochemical Measurements

Filter paper was cut into discs of approximately 8 mm using a hole puncher and a series of 20 μL aliquots of reconstituted MT solutions were added to the discs to determine the optical concentration. The discs were kept in the fridge and allowed to dry under nitrogen to prevent oxidation. Once dry, the discs were placed on a DropSens electrode. The integration of the protein laden disc and SPCE has been described in detail elsewhere [37]. For the Hg^2+^ experiments measurements were taken with an incubation time of 2 min to allow for equilibration. While the reaction of apo-MT with mercury is rapid, the equilibration time is needed to allow for displacement of the Zn/Cd bound to the protein on the paper disc.

For the As^3+^ experiments, HCl was added to the metal solutions to adjust the pH to 2.0. This was done to remove Zn^2+^ and Cd^2+^ still bound to MT adsorbed onto the paper disc. The equilibration time was longer, up to 30 min to allow for complete reaction as the kinetics of As^3+^–MT binding is known to be slow at room temperature [26].

Anodic stripping voltammetry (ASV) measurement parameters for both metal detection experiments were as follows: deposition potential −350 mV; deposition time 150 s; scanning range between −0.2 and +0.3 V; scanning rate 100 mV/s; step W 20 ms; step H 2 mV; eChem stripping linear mode.

## 3. Results and Discussion

**Figure 1:**
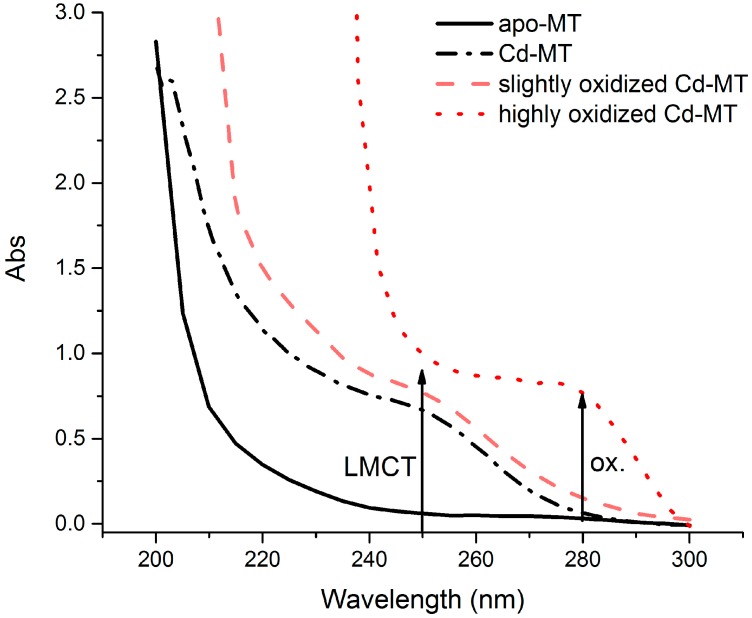
Far UV absorption spectra of metallothionein (MT) used to monitor oxidation of thiols. Apo-MT (solid black line), Cd-MT (dashed black line) are both fully reduced with no thiol oxidation. Aerated MT solutions are shown to illustrate the oxidation: minimal oxidation (dashed pink line) and significant oxidation (dotted red line). The ligand-to-metal charge transfer (LMCT) band and the oxidized thiol absorbance are highlighted with arrows.

The quality of the MT protein adsorbed on to the paper discs was checked first by ESI-MS prior to and after lyophilization to ensure purity. The protein was examined again prior to disc preparation by far UV absorption spectroscopy to monitor for oxidation of the thiols. UV spectroscopy is a fast, non-destructive technique for determining MT quality and concentration. The ligand-to-metal charge transfer (LMCT) absorption at 250 nm was used to calculate protein concentration. For use in the biosensor, it was crucial that there was little (<0.15 abs) absorbance at 280 nm which would indicate the presence of oxidized thiols (Figure 1). 

**Figure 2:**
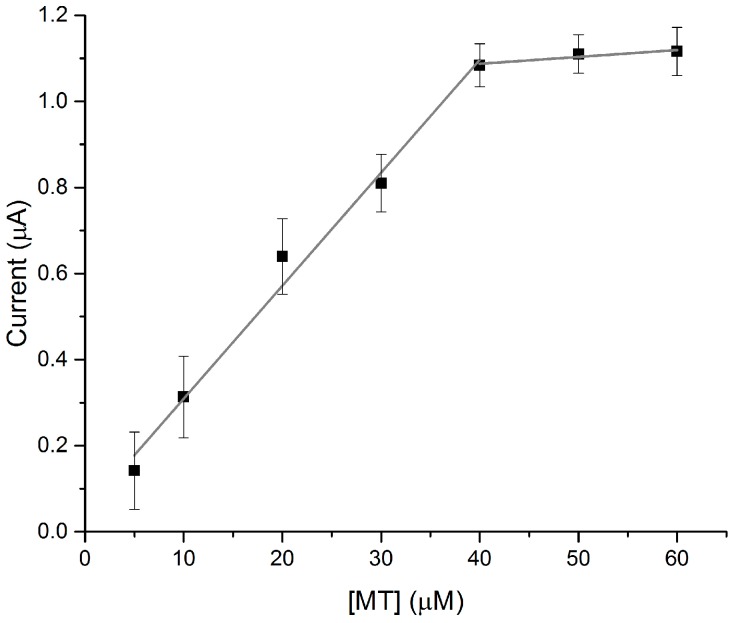
Influence of paper disc MT-loading on the peak heights of the S_Cys_ responses at +0.22 V in the electrochemical scans. The MT concentrations of each of the 20 μL aliquots added to the paper discs are plotted on the x-axis (*n* = 3).

In the preparation of the MT-loaded discs, it was determined that solutions of 40 μM gave the best signal response (Figure 2). Adsorption of additional aliquots of MT solution did not increase the signal response significantly, nor did the addition of higher concentrations of MT. This addition of 40 μM concentration of MT to the paper discs results in approximately 8 μg of protein being loaded in total. It should be noted that MT contains 20 cysteiene residues and is stored bound to Cd^2+^ so the thiol and Cd^2+^ concentration loaded on to the disc is 20 and 7 times larger, respectively.

**Figure 3:**
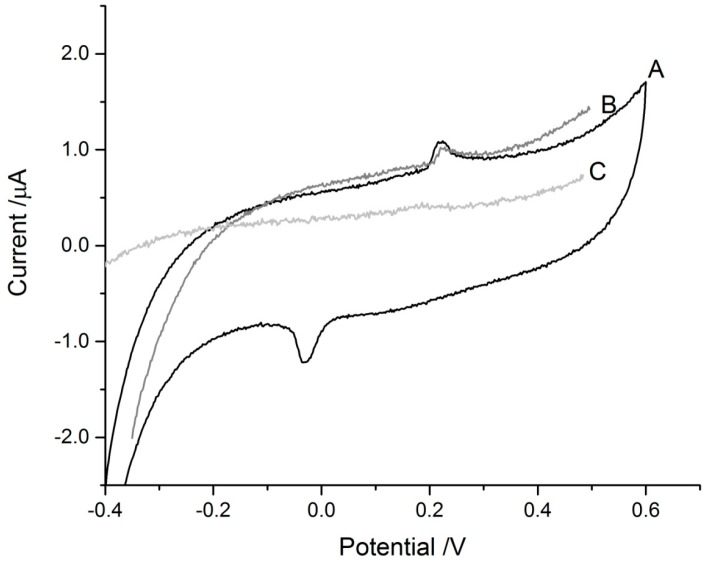
Representative electrochemical measurements of MT-loaded and blank-paper discs on SPCEs with 25 μL of deionized water added. (**A**) Cyclic voltammetry (CV) scan of MT-adsorbed disc; (**B**) anodic stripping voltammetry (ASV) scan on MT-adsorbed disc; and (**C**) ASV scan of blank paper disc. The ASV and CV scans of the blank paper disc were similar and showed no distinct peaks.

The blank discs on the SPCEs did not show any signals in the scans performed (Figure 3). The MT-adsorbed discs gave signals slightly above +200 mV in both ASV and CV scans, likely due to the presence of free thiols in the protein. There was also a reduction peak around −50 mV in the CV scan which corresponds to the cysteinyl thiols in MT. The heights of the peaks are relatively small and this is due to the presence of Zn/Cd in the isolated, lyophilized protein masking the thiol signal. The presence of these bound metals is essential to the stability of the protein for longer periods of storage, as apo-MT degrades more rapidly and is prone to oxidation [38]. Compared to apo-MT, Cd/Zn-MT can be stored as a frozen solution or lyophilized and reconstituted after many years of storage without significant degradation.

### 3.1. Arsenic Detection Using ASV

**Figure 4:**
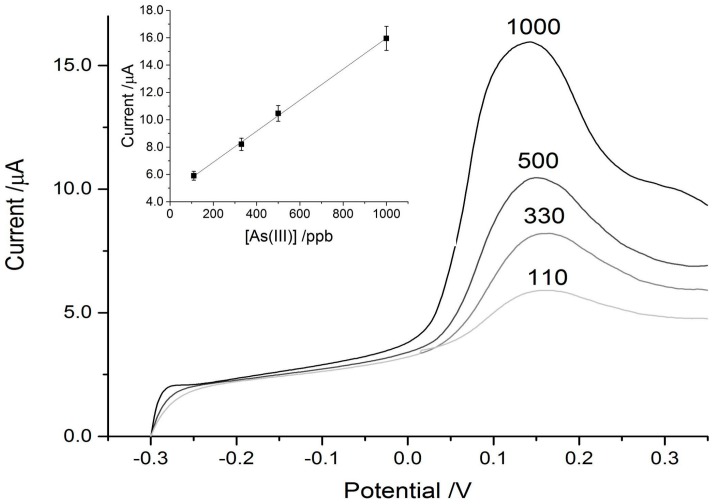
Typical ASV scans using blank paper discs on screen-printed carbon electrodes (SPCEs) with 25 μL of arsenic solutions of varying concentration added. The arsenic concentrations are indicated above their respective trace. Inset in the top left is the linear response curve for the blank disc set-up.

**Figure 5:**
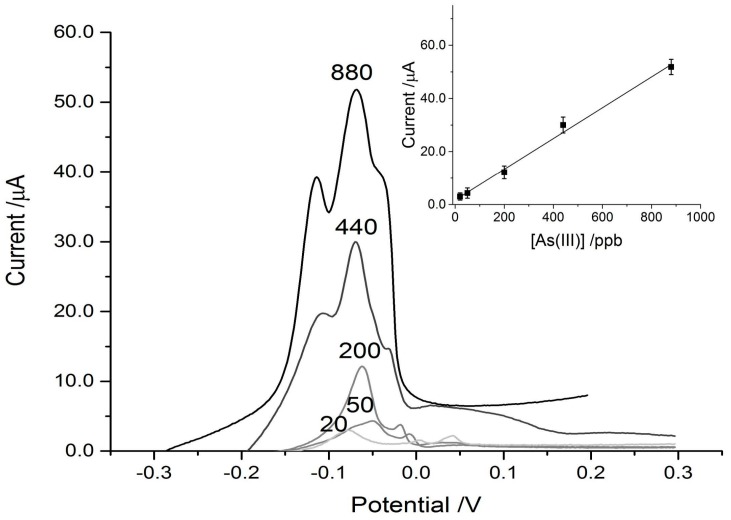
Representative ASV scans of MT-adsorbed paper discs with 25 μL droplets of varying arsenic concentrations added. The concentrations are labeled above their respective traces. Inset in the top right is the linear response curve for the MT-disc set-up.

The paper discs on the SPCE connected to the DropSens device, coupled with the eChem software was able to detect As^3+^ reasonably well in the control experiments, achieving a strong signal down to a concentration of 100 ppb. The limit of detection (LOD) (3S/N) calculated for the control paper disc on the SPCE was 69 ppb. For reference, the WHO recommended limit for arsenic in drinking water is 10 ppb. When the MT was physically adsorbed on to the paper discs placed over the SPCEs, the sensitivity was increased and the signal intensity amplified greatly. This can be seen in Figure 4 and Figure 5, where the MT disc achieved a three-fold signal enhancement, which results in a detection limit below 20 ppb As^3+^. LOD (3S/N) was calculated to be 13 ppb for the MT-adsorbed discs on the SPCEs. While this is still higher than the WHO recommended limit for drinking water, it is low enough to potentially be useful in screening for arsenic contamination in high risk areas. For example, the Cambodian recommended limit is 50 ppb with some wells having concentrations as high as 3.5 ppm (to emphasize the danger, 3500 ppb) [39]. In Bangladesh, 43% of wells have concentrations exceeding 50 ppb [17]. The incorporation of the cysteine-rich metallothionein pushed the LOD to concentrations below that typically found in areas of concern.

In addition to an increase in signal intensity, the interactions between the arsenic and MT-disc shifted the peak intensity to a more negative potential. The control disc with no MT adsorbed showed As^3+^-dependent peaks at approximately +160 mV (Figure 4) which is very similar to that reported in the literature with more sensitive electrodes [40,41,42]. The potential of As^3+^-stripping in the MT-modified disc scans was shifted to approximately −60 mV and this type of shift is consistent with coordination by thiol [43] and protein modified electrodes [44]. Both the control and experimental traces had high degrees of linearity over the range tested (Table 1).

### 3.2. Mercury Detection Using ASV

In addition to As^3+^, which selectively binds to MT at low pH, we sought to leverage the higher affinity that MT generally has for Hg^2+^ over Zn^2+^ and Cd^2+^, as similar strategies have been previously tested for MT-based metal detection [45]. Since the affinity for Hg^2+^ is higher than that of the native metals bound to MT, Hg^2+^ will displace these metals [46]. In addition, both zinc and cadmium require more negative plating potentials than were applied during this experiment, limiting their interference in this device. Upon lowering the pH for arsenic detection or the displacement by mercury, the interferent (Cd/Zn) concentration is approximately 280 μM.

**Figure 6:**
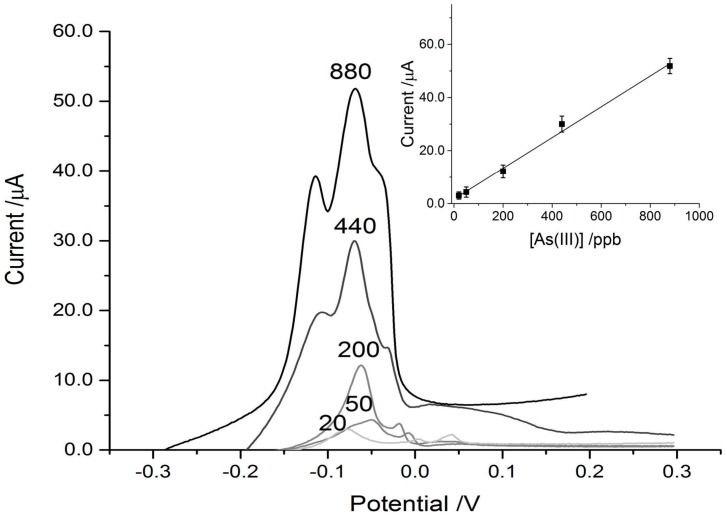
Representative ASV scans of blank paper discs on SPCEs with 25 μL aliquots of Hg^2+^ solutions of concentrations 20, 50 and 200 ppb. The concentration corresponding to each trace is labeled near the peaks at +0.1 V. Inset in the top left corner is the linear fit of the control data (n = 3).

**Figure 7:**
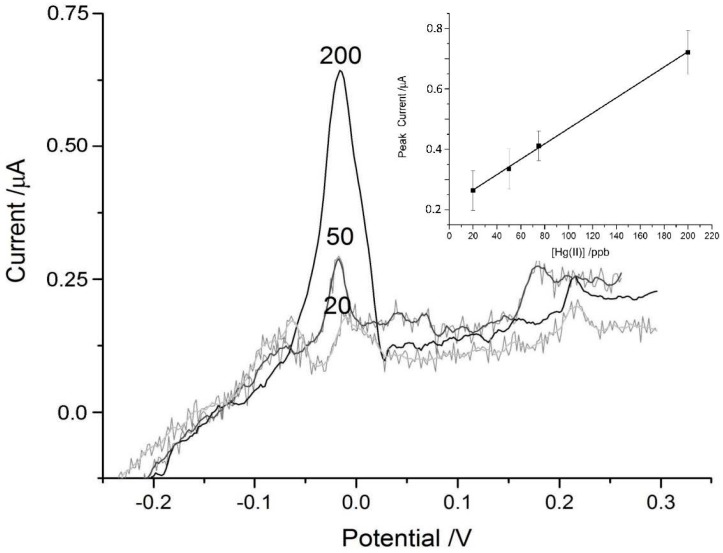
Representative ASV scans for MT-loaded discs with 25 μL drops of diluted Hg^2+^ standards with concentrations of 20, 50 and 200 ppb. The concentration is indicated on the graph near the Hg^2+^ peaks around −0.02 V. The peaks near +0.2 V correspond to the thiols of metallothionein. Inset in the top right corner is the linear fit of the data (*n* = 3).

Below 20 ppb detection of Hg^2+^ became impossible even with the enhancement from the MT-adsorbed discs. A comparison of Figure 6 and Figure 7 show the approximately three-fold enhancement of the peak current achieved by the MT-disc. The WHO guideline upper limit for mercury in drinking water is six parts per billion, meaning the device is not achieving the sensitivity required to detect such low concentrations, especially in more natural samples with organic interferents. However, it may be more useful in monitoring area suspected of contamination which typically have higher concentrations that the recommended limit [47]. The LOD (3S/N) for the MT-modified paper disc was 45 ppb and 120 ppb for the blank paper disc.

The peak current for the Hg^2+^ signals was shifted to a more negative potential (Figure 7) similar to the shift in the As^3+^ signal seen in Figure 4 and Figure 5. The peak seen around +200 mV in the experimental trace is from the thiols in the protein and was also observed in the control tests where deionized water was added to the MT-discs (Figure 3).

The linearity over the range of mercury concentrations typically found in water contaminated by industry is adequate since even polluted waters have concentrations up to 200 ppb [47,48,49]. While the sensitivity of the device is not ideal for sensing lower concentrations of Hg^2+^ typical of non-industrially polluted waters, we have demonstrated the versatility in our approach in incorporating a protein known to interact with many soft metals and metalloids that cause environmental health problems. MT is able to enhance the signal of both metals tested due to its promiscuity of metal binding and lack of discriminatory metal binding sites.

The potential of MT-incorporated devices for sensing a wide range of metals is summarized in Table 2, which lists examples of various metallothioneins being used to enhance detection methods. The advantage of this strategy is that MT can enhance methods with specific advantages such as specificity, low detection limit, portability or low-cost. In our paper disc/SPCE sensor, we enhance a ‘green’ paper matrix with MT to produce a more sensitive As^3+^/Hg^2+^ sensor. The low-cost and environmentally friendly design of the sensor is greatly enhanced with the protein modification because the detection limit pre-MT adsorption is much higher than what would be considered acceptable.

By modifying the conditions under which the ASV scans are run, we can use MT as an effective and selective pre-concentration agent for both As^3+^ and Hg^2+^. Thus, the same device could be used for different assays depending on the type of contamination suspected in the given area. Table 2 shows a summary of the different metals sensed by a number of MT-based biosensors. It is likely a simple MT biosensor would be able to measure all the metals listed in Table 2, but slightly different strategies or configurations might have to be used. By using different MT isoforms [50] or a rational design approach [51,52] to increase the binding affinity for certain metals, a device could be customized for any soft metal. Another strategy would be to incorporate apo-MT to eliminate the need for the sensed metal to have a much higher affinity than the endogenous metals (Cd/Zn). This would pose additional engineering constraints due to the high oxygen sensitivity of metal-free metallothionein and would require more extensive sample preparation to remove dissolved oxygen [53,54,55].

## 4. Conclusions

Adsorbing the cysteine-rich protein metallothionein on to paper discs provided a simple, specific, sensitive and inexpensive sensor for the toxic metals As^3+^ and Hg^2+^. Metallothionein incorporation allowed the SPCE/paper disc system to sense As^3+^ at low enough concentrations to be potentially useful in environmental monitoring. This signal enhancement is likely due to pre-concentration effects of the MT-metal coordination. Our work shows that even simple physical adsorption of the protein onto an inexpensive SPCE/paper disc can dramatically increase the signal associated with a metal of interest without the need for costly coupling reagents or expensive and complicated set-ups.

## Figures and Tables

**Table 1:** Equation of linear regressions for As^3+^ and Hg^2+^ determinations.

Experiment	Linear Regression Equation	R^2^
As^3+^ control	y = 1.135 × 10^−8^(x) + 4.621 × 10^−6^	0.9977
As^3+^ MT-discs	y = 5.999 × 10^−8^(x) + 1.425 × 10^−6^	0.9860
Hg^2+^ control	y = 5.494 × 10^−10^(x) + 1.755 × 10^−7^	0.9843
Hg^2+^ MT-dics	y = 2.545 × 10^−9^(x) + 2.132 × 10^−7^	0.9993

**Table 2:** Summary of MT-incorporated biosensors.

Metal	Method/Electrode Type	Modifiers and Extra Components	Detection Limit	Reference
Cd, Zn, Ni	SPR	MT on carboxymethlyated chips	1–2.25 ppm	[21]
Cd, Zn	HDME	TCEP	5.6 ppb	[41]
Cd	Fluorescence	Zn-Chelex resin, Rh-labelled MT	50 ppb	[36]
Pt (cisplatin)	HDME	TCEP	10 ppb	[23]
Ag	Carbon paste	anti MT-antibodies	0.05 ppb	[25]
Pd	HDME	TCEP	10 ppb	[42]
As	Paper disc/SPCE	MT-adsorption	13 ppb	This work
Hg	Paper disc/SPCE	MT-adsorption	45 ppb	This work
Hg, Cu, Zn, Cd	Modified gold electrode	Coupling agents, bacterial MT, continuous flow set-up	1 × 10^−15^ M	[43]
