# Erratum: Irvine, G.W.; Tan, S.N.; Stillman, M.J. A Simple Metallothionein-Based Biosensor for Enhanced Detection of Arsenic and Mercury. *Biosensors* 2017, *7*, 14

**DOI:** 10.3390/bios7020018

**Published:** 2017-05-05

**Authors:** Biosensors Editorial Office

**Affiliations:** MDPI AG St. Alban-Anlage 66, 4052 Basel, Switzerland; biosensors@mdpi.com

The *Biosensors* Editorial Office wishes to report the following erratum to this paper [1]. In the paper, Figure 6 is the same as Figure 5. The correct version of Figure 6 is as follows: 

The error was introduced during production. We apologize for any inconvenience caused to the readers by this mistake. The manuscript will be updated and the original will remain online on the article website.

## Figures and Tables

**Figure 6:**
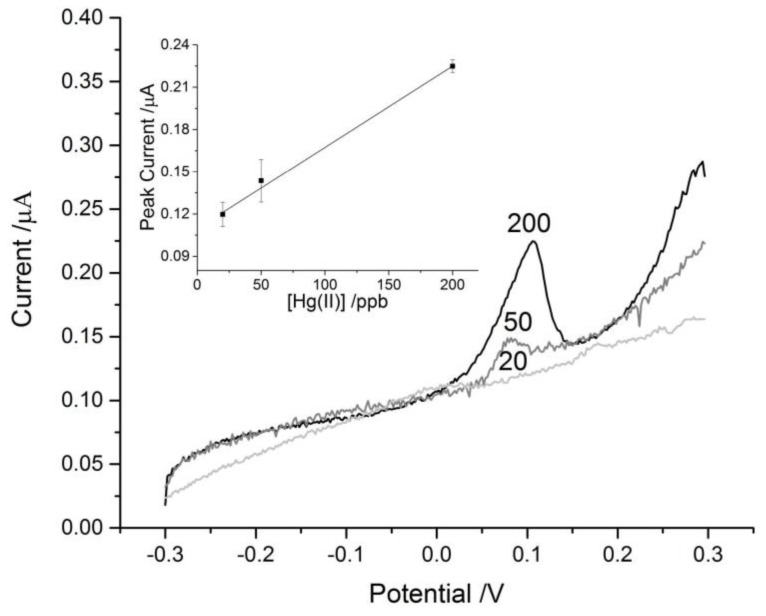
Representative ASV scans of blank paper discs on SPCEs with 25 μL aliquots of Hg^2+^ solutions of concentrations 20, 50 and 200 ppb. The concentration corresponding to each trace is labeled near the peaks at +0.1 V. Inset in the top left corner is the linear fit of the control data (*n* = 3).
